# Significance of 8-iso-PGF_2α_ in cardiovascular diseases

**DOI:** 10.1016/j.ahjo.2026.100782

**Published:** 2026-04-09

**Authors:** Paola Simeone, Rossella Liani, Stefano Lattanzio, Maurizio Frezza, Margherita Alfonsetti, Francesco Cipollone, Francesca Santilli

**Affiliations:** a Department of Medicine and Aging Sciences, Center for Advanced Studies and Technology (CAST), University of Chieti, 66100, Chieti, Italy

**Keywords:** Oxidative stress (OS), 8-iso-PGF_2α_, Lipid peroxidation, Cardiovascular diseases

## Abstract

Oxidative stress (OS), derived from an imbalance between reactive oxygen species (ROS) accumulation and impaired antioxidant defense, is a recognized cause of atherothrombosis, through a complex interaction between low-grade inflammation and platelet activation.

Lipid peroxidation, as reflected by the urinary excretion of 8-iso-Prostaglandin F2α (8-iso-PGF_2α_), is central in the pathogenesis of atherosclerosis. This biochemical abnormality has been observed in patients with cardiovascular risk factors, including diabetes mellitus, obesity, cigarette smoking, hypercholesterolemia, hypertension, atrial fibrillation, and in clinical settings associated with aging, such as acute and chronic cardiovascular diseases and chronic kidney disease.

Despite the treatment with acetylsalicylic acid or with any other antithrombotic drugs, patients may undergo recurrent events due to the complex nature of atherothrombosis. A large body of evidence supports the relationship between OS and less-than-expected response to aspirin. Several disease-modifying agents and antioxidant supplementation, as well as modulation of the primary metabolic abnormalities driving lipid peroxidation, have been shown to reduce urinary 8-iso-PGF_2α_ excretion.

Overall, these observations pave the way for potential therapeutic approaches able to target these mechanisms, resulting in the reduction of atherothrombosis progression. This will be an overview of the significance of 8-iso-PGF_2α_ in the pathogenesis of atherothrombosis and as a potential mechanism-based biomarker of cardiovascular events.

## 1 Introduction

Oxidative stress (OS) is a condition of imbalance where the overproduction of reactive oxidative species (ROS) overwhelms the antioxidant capacity of the biological system [1]. It is involved in several diseases such as arthritis, atherosclerosis, Alzheimer's disease, cancer, diabetes, hypertension and inflammation [2]. ROS could damage nucleic acids (DNA and RNA), proteins and lipids and contribute to inducing and accelerating disease. Direct measuring of ROS in laboratory practice is rather difficult due to their primary characteristic, i.e., highly reactive species with consequent short life. For this reason, OS is measured by evaluating the damage induced by ROS on specific compounds. The formation of isoprostanes (IsoPs) such as urinary8-iso-Prostaglandin F 2α (8-iso-PGF_2α_), *in vivo* can be monitored reliably and non-invasively by analytical approaches that provide us with information on the state of OS in humans. Furthermore, these molecules are stable in their biochemical structure and this peculiarity contributes to their designation as dependable biomarkers [2].

IsoPs have been regarded as a) potential and interesting biomarkers of the level of OS *in vivo* [3], [4]; b) mediators of cardiovascular (CV) and non-CV diseases; c) responsible for the increase in CV risk [3], d) link between OS and both platelet activation and smooth muscle cell proliferation in CV diseases [4].

## 2 Pathophysiology

Free radicals generated primarily from oxygen are involved in the pathogenesis of different human pathologies [5]. The free radicals, both ROS and reactive nitrogen species (RNS), are produced from various endogenous sources such as peroxisomes, mitochondria, endoplasmic reticulum, phagocytic cells and exogenous sources like pollution, alcohol, tobacco smoke, heavy metals, industrial solvents transition metals, pesticides, certain drugs like halothane, paracetamol, and radiation [5]. The overproduction and the imbalance between production/elimination of ROS and RNS define the state known as oxidative stress (OS) [6].

ROS include superoxide anion radical (O_2_•−), hydrogen peroxide (H_2_O_2_), hydroxyl radical (•OH), and singlet oxygen (1O_2_), while RNS include nitric oxide (NO•), peroxynitrite (ONOO−), and nitrogen dioxide (NO_2_) [6]. Under normal physiological conditions these reactive species pose no threat to the body [168], [169], but if they are not adequately removed, the resulting OS participates in the formation of atherosclerotic plaques increasing the risk of type 2 diabetes mellitus (T2DM) and coronary heart disease (CAD) [7].

Sources of ROS production are manifold, but mitochondrial activity and its electron transport chain in aerobic respiration is considered the primary source [8], [9].

One of the major targets of free radical injury is peroxidated lipids. The presence of polyunsaturated fatty acids (PUFA) causes lipid peroxidation of Arachidonic acid (AA) which is highly sensitive to ROS-induced oxidative damages or free radicals, generating a wide variety of derivatives, known as IsoPs [8].

The interest has been directed to the F2-IsoPs [10], because, not being influenced by the diet lipid content, their evaluation in biological fluids (particularly in urine) and in exhaled breath condensate is a reliable index of their total levels [9]. Thus, F2-IsoPs are a family of biologically active lipid mediators, formed in situ at the phospholipid domain of cell membranes by the free radical lipid peroxidation of AA or circulating LDL particles via a non-cyclooxygenase dependent pathway [11]. The interest in F2-IsoPs raised for two main reasons: they can be ligands of prostaglandin receptors exhibiting biological activity as other AA metabolites [12], and are associated with the OS status [13], [14]. In fact, Roberts and Morrow reported that IsoPs are major products of radical-mediated lipid oxidation in vivo, and have been widely recognized as reliable oxidative biomarkers [13], [14]. Numerous investigations have shown that obese patients and/or diabetes mellitus (DM) patients have high circulating and urinary levels of F2-IsoPs [15]. Moreover, a correlation between body mass index (BMI) and levels of IsoPs in urine has been reported by the Framingham study [16].

From the peroxidation of AA, four positional F2-IsoP isomers are generated and each of them can be composed by eight racemic diastereomers.

8-iso-PGF_2α_ (also called 8-epi-PGF_2α_) is characterized by specific biological activities as pulmonary and renal vasoconstrictor [17], [18], mediator of hepatorenal syndrome and pulmonary oxygen toxicity [18] and in T2DM, correlates with the rate of thromboxane (TX) A_2_ biosynthesis. We and other hypothesized that the increased OS observed in disease, results in an increased 8-iso-PGF_2α_ generation and this molecule could participate to platelet activation [19].

Indeed, 8-iso-PGF_2α_, together with platelets agonists, mediates aggregation, and this synergistic effect reflects the conditions existing in the peculiar microenvironment of atherosclerosis. Exposition to an increasing amount of 8-iso-PGF_2α_ with minimal sub-threshold concentration of collagen, ADP, AA and some chemical analogs of TXA_2_ determines a dose-dependent and irreversible aggregation of platelets [20]. Pharmacological inhibition of 8-iso-PGF_2α_-mediated platelet response by thromboxane-receptor antagonists provides an indirect proof that 8-iso-PGF_2α_ is an agonist of thromboxane receptor [21].

A study by FitzGerald et al. [22] demonstrated that IsoPs (8-iso-PGF_2α_ and iPE_2_-III) exerted their effects on platelets and vascular tone *in vivo* by acting as membrane ligands of the TP rather than via a distinct IsoPs (iP) receptor. TPs activation, mediated by iPs, may be relevant in pathologies in which COX activation and OS coincide, such as in tissue ischemia-reperfusion and atherosclerosis.

Indeed, an additional work demonstrated that 8-iso-PGF_2α_ can act on platelets by two separate binding sites, a stimulatory (TP-dependent) and an inhibitory (cAMP-dependent) pathway [12].

The correlation between of 8-iso-PGF_2α_ formation rate and thromboxane levels in obese women [23], in patients with hypercholesterolemia [24], T1DM [25] and T2DM [26], or homozygous homocystinuria [27], inflammatory bowel disease (IBD) [28], chronic kidney disease (CKD), suggests a role for low-grade inflammatory condition, connected with these metabolic disorders, as the major trigger of thromboxane (TX)-dependent platelet activation. These phenomena can be mediated by an enhanced lipid peroxidation [29].

## 3 Laboratory assay and methods

In the field of research, one of the major obstacles to evaluate the state of OS in humans has been the lack of reliable methods for the assay of free radicals [30]. The quantification of one of the products generated by a high condition of OS, IsoPs, has allowed us to investigate the extent of systemic lipid peroxidation.

F2-IsoPs may be found in a variety of biological fluids and tissues, including the liver, kidney, stomach, brain, lung, vascular tissue, muscle, and heart [31]. This allows us to investigate the production of these compounds locally in oxidant damage sites [31]. IsoPs measurement in body fluids is far more feasible and less intrusive in human investigations than assessing these substances in organ tissue. F2-IsoPs quantification in plasma, platelet or urine provides a highly precise and accurate index of OS, basing on available data [32], [33]. A potential limitation of measuring F2-IsoPs is that they can be generated ex vivo in plasma and other biological fluids in which lipids that contain arachidonyl are present. When biological fluids or tissue are left at room temperature or stored at −20 °C, this unfavorable event can occur [31].

To prevent F2-IsoPs from being artificially generated *ex vivo*, appropriate precautions must be taken. Indeed, it has been found that F2-IsoPs is not formed ex vivo a) if biological fluids or tissues are processed immediately, b) if supplementation with indomethacin is undertaken in vacutainers for blood collection to give a final concentration of 10 μM. Indomethacin is able to impede *ex-vivo* formation of F2-IsoPs and other eicosanoids which have the potential to interfere with the EIA assay [34], c) if antioxidants, such 1 mmol/L of 4-hydroxy-Tempo, are added immediately to urine and stored at −80 °C until extraction [35], d) if agents such as free radical scavenger butylated hydroxytoluene (BHT), and/or the reducing agent triphenylphosphine are added to organic solvents during the extraction and hydrolysis of phospholipids [31].

Measurement of 8-iso-PGF_2α_, one of the most abundantly produced compounds and recognized as a potent vasoconstrictor, can be performed in platelet [33], plasma or urine samples [36].

Urinary and platelet 8-iso-PGF_2α_ levels correlation indicates that urinary 8-iso-PGF_2α_ excretion may reflect, in part, platelet 8-iso-PGF_2α_ production. An interventional study strengthened this hypothesis demonstrating that up and down regulations of platelet 8-iso-PGF_2α_ levels were associated with urine 8-iso-PGF_2 α_ excretion rate [33].

The presence of a wide variety of well-known interfering materials could be avoided if urinary samples undergo a chromatographic extraction process before being analyzed by immunoassay [37].

Different investigators, including FitzGerald et al., developed a variety of mass spectrometric assays [34]. Most of them require C18 solid phase extraction, TLC purification, and chemical derivatization. In general, these various methods are comparable. The high sensitivity and specificity of mass spectrometry, compared to other methods, allows performing quantification in the low picogram range. GC–MS has been used as the primary tool to measure 8-iso-PGF_2α_ levels, but its limitation is its limited ability to analyze large amounts of clinical material in a short time [38].

Enzyme immunoassay (EIA) and a radioimmunoassay (RIA) were developed to detect and quantify 8-iso-PGF_2α_ in urine samples (IC50, 8 and 24 pg/mL, for EIA and RIA, respectively) and they were validated with a negligible cross-reactivity with another prostaglandin. 8-iso-PGF_2α_ quantification in urine, by immunoassays (EIA; RIA), were validated using numerous antibodies and were further compared with GC/MC. RIA is used for the analysis of human, rabbit, porcine and rat plasma, and urine 8-iso-PGF_2α_ [38].

The ability to readily detect F2-IsoPs in fresh plasma and urine established the notion of their formation in vivo. Herein, in animal models exposed to oxidant injuries (i.e. administration of CCL or diquat to selenium-deficient rats) the F2-IsoPs levels resulted 200-fold increased [35].

**Fig. 1:**
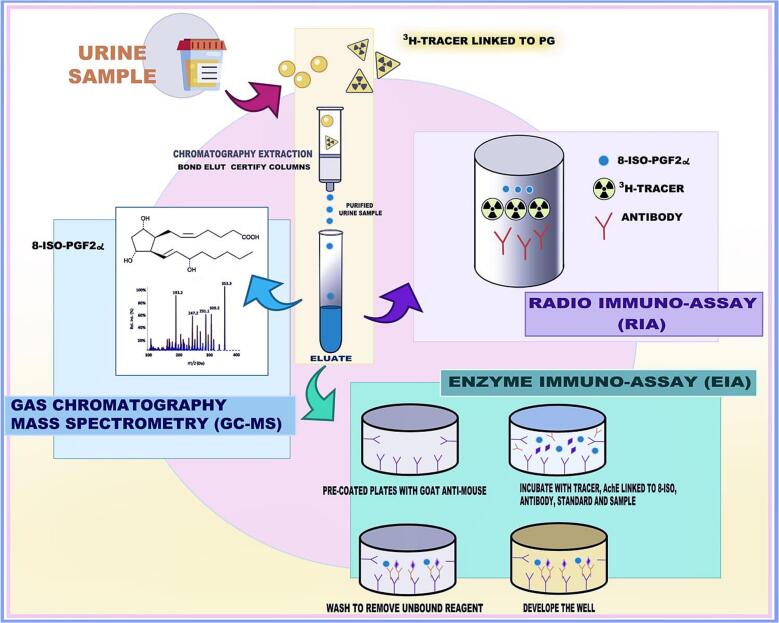
Laboratory assay and methods to evaluate Urinary 8-iso-PGF_2α_. The measurement of 8-iso-PGF_2α_ requires purifications of the urine sample by a specific chromatography technique based on the use of Bond-Elut Certified columns. The purified sample (eluate) can be dosed with different methods such as Gas Chromatography-Mass Spectrometry (GC–MS), RIA and EIA. GC–MS, the “gold standard” technique combines the features of gas-chromatography and mass spectrometry to detect a variety of substances within a test sample. RIA is a sensitive *in vitro* assay used to detect levels of specific molecules using antibodies and a known radioactive binded antigen. The antibody binding and enzymatic detection are combined to evaluate molecular targets, and it is based on the competition between the molecule of interest and an enzyme-conjugated version of the same molecule (8-iso-PGF_2α_-tracer) and, after incubation, the enzymatic reaction product, reflected by absorbance values, is read using a spectrophotometer.

In order to evaluate EIA inter-assay reproducibility, the same urine frozen in different tubes underwent solid phase extraction and thin-layer chromatography and using several antisera to further compare the results with those obtained with GC–MS [37]. Healthy subjects excreted 25 ± 12 ng of 8-iso-PGF_2α_/mmol creatinine and no circadian variation, over three consecutive 8 h collection times, was observed. Moreover, different results demonstrated that the increased 8-iso-PGF_2α_ excretion is age-related. Nonsteroidal anti-inflammatory drugs treatment did not affect the urinary excretion of 8-iso-PGF_2α_. It is reasonable to state that 8-iso-PGF_2α_ is the “gold standard” to detect lipid peroxidation in vivo (Fig. 1) [29].

However, in light of the high cost of the measurement and the possibility that it would need gas/liquid chromatography combined with mass spectroscopy methods (HPLC/GC–MS), the use of F2-IsoPs as biomarkers remains extremely limited in clinical practice. Furthermore, if the sample is not treated correctly, as previously mentioned, the high frequency of AA autoxidation increases the likelihood of inaccurate evaluation of F2-IsoPs [14]. Therefore, in assessing F2-IsoPs in clinical settings, these limitations must be taken into account. Interestingly, 8-iso-PGF_2α_, an isomer obtained from F2-IsoPs, appears to be the most relevant biomarker as it exhibits greater stability, sensitivity, and specificity when used as a marker of OS.

## 4 8-iso-PGF_2α_ and atherothrombosis

Lipid peroxidation is an important player in the development of atherosclerosis. LDL-C oxidation increases their availability for uptake by macrophages, which is a primary factor in the development of plaque, vascular inflammation, and foam cell transformation [39]. Concentrations of 8-iso-PGF_2α_ ranging from 1 nmol/L to 1 μmol/L cause an increase in inositol phosphates, calcium release from intracellular reserves, and dose-dependent platelet shape change [40]. F2-IsoPs, specifically 8-iso-PGF_2α_, can modulate both platelet and vascular function [41] amplifying the aggregation response in the presence of low levels of platelet agonists [21]. When compared to healthy vessels, IsoPs result increased in human atherosclerotic plaque, in patients with carotid endarterectomy [42]. 8-iso-PGF_2α_ levels were also assayed by immunohistochemistry (IHC) in lipid-rich atherosclerotic lesions mostly associated with smooth muscle cells and macrophages, thus supporting the idea that the evaluation of IsoPs may provide a quantitative manifestation of OS in human atherosclerotic pathology [43]. By promoting platelet activation, enhancing adhesion, raising intracellular calcium concentration, and decreasing nitric oxide (NO) antagonist activity toward its antiadhesive and antiaggregatory effects, 8-iso-PGF_2α_ may be considered as a connection between lipid oxidation and thrombotic complications in atherosclerotic disease [40]. The indices of remodelling and levels of 8-iso-PGF_2α_ showed a substantial correlation in individuals suffering from ischemic chronic heart failure [44].

### 4.1 Acute coronary syndrome (ACS)

Acute coronary syndromes are characterized by a marked increase in oxidative stress, which plays a central role in endothelial dysfunction, plaque destabilization, and thrombotic complications [45]. Evidence from clinical studies consistently demonstrates that 8-iso-PGF_₂α_ levels are significantly elevated in patients with ACS, supporting its role as a reliable biomarker of in vivo lipid peroxidation [45]. In particular, patients with unstable angina exhibit substantially higher urinary excretion of 8-iso-PGF₂α compared to those with stable angina or healthy controls, indicating that enhanced oxidative stress is specifically associated with plaque instability rather than myocardial ischemia per se. This concept is further supported by the lack of significant changes in 8-iso-PGF_₂α_ levels during transient ischemia induced by stress testing [46], [47].

Additional evidence shows that 8-iso-PGF_₂α_ levels increase markedly in the context of myocardial infarction and during ischemia–reperfusion injury, such as after thrombolysis or coronary revascularization procedures, suggesting that oxidative mechanisms are amplified during acute vascular injury [48].

**Fig. 2:**
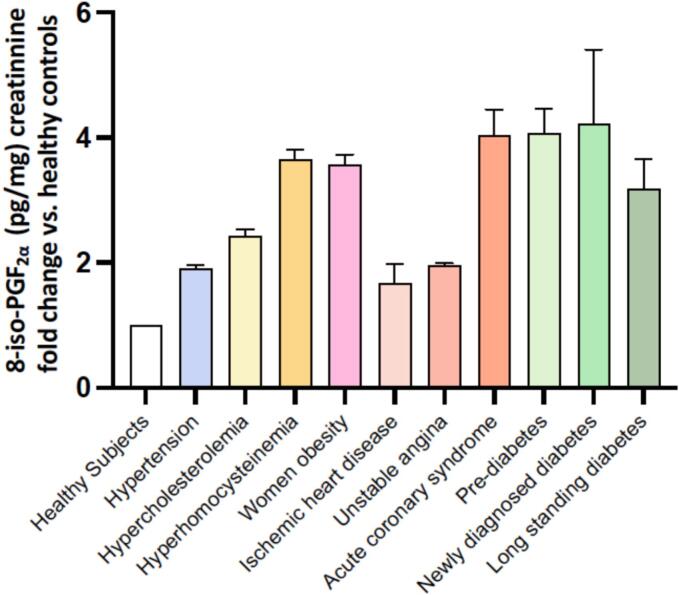
Urinary-8-iso-PGF_2α_ levels in healthy subjects and in cardiovascular diseases. Median (interquartile range) urinary excretion rates of urinary 8-iso-PGF_2α_, evaluated by previously described and validated immunometric method [38], in clinical settings characterized by high cardiovascular risk. 8-iso-PGF_2α_ levels in: healthy subjects [51], hypertension [51], hypercholesterolemia [33], hyperhomocysteinemia [52], women obesity [24], ischemic heart disease [50], unstable angina [33], acute coronary syndrome [50], pre-diabetes [53], newly-diagnosed diabetes [53], long standing diabetes [53]. Enhanced platelet activation in circulating blood and in a microvascular injury model was linked to high levels of 8-iso-PGF_2α_ in ACS, indicating a systemic effect of OS related to myocardial infarction on local hemostasis.

Importantly, 8-iso-PGF_₂α_ appears not only as a marker but also as a potential mediator of thrombotic risk. Indeed, its levels have been shown to correlate with thromboxane biosynthesis, supporting a link between lipid peroxidation and platelet activation. In this context, elevated urinary 8-iso-PGF_₂α_ has been identified as a predictor of residual thromboxane-dependent platelet activation in ACS patients, even in the presence of antiplatelet therapy [49] (Fig. 2).

Taken together, these findings highlight the dual role of 8-iso-PGF_₂α_ in ACS, reflecting both the extent of oxidative stress and its contribution to platelet-driven thrombotic processes, thereby reinforcing its potential clinical relevance as a mechanism-based biomarker.

### 4.2 Stroke

Oxidative stress plays a central role in the pathophysiology of ischemic stroke, contributing to both thrombus formation and its subsequent stabilization. Reactive oxygen species (ROS), generated by platelets, leukocytes, and endothelial cells, promote endothelial dysfunction, enhance platelet activation, and modulate fibrin structure, thereby facilitating thrombus development and persistence [50]. Among oxidative biomarkers, 8-iso-PGF_₂α_ has emerged as a key mediator linking ROS generation to platelet activation. Experimental evidence indicates that ROS-driven production of 8-iso-PGF_₂α_ can activate glycoprotein IIb/IIIa, thereby enhancing platelet recruitment and aggregation. This mechanism is further supported by studies in patients with NADPH oxidase deficiency, in whom reduced 8-iso-PGF_₂α_ formation is associated with impaired platelet activation, while exogenous administration restores platelet recruitment [51].

Clinical studies consistently demonstrate elevated levels of F2-isoprostanes, including 8-isoPGF_₂α_, in patients with ischemic stroke compared to controls. Both plasma and urinary measurements have been associated with increased stroke risk, independently of traditional cardiovascular risk factors. Notably, higher urinary 8-iso-PGF_₂α_ levels have been linked to an increased likelihood of stroke events in population-based studies, suggesting a potential role in risk stratification [33].

Taken together, these findings support a dual role of 8-iso-PGF_₂α_ in ischemic stroke, acting both as a biomarker of oxidative stress and as an active contributor to platelet-mediated thrombosis. This reinforces its potential relevance in identifying patients at higher thrombotic risk and highlights oxidative stress as a possible therapeutic target in cerebrovascular disease.

### 4.3 PAD

Lower-extremely Peripheral Artery Disease (PAD) is a significant site of atherosclerosis, associated with significant deterioration in multiple arterial regions. In fact, the risk of both coronary and cerebral vascular disease is predicted by PAD.

The development of atherosclerotic vascular disorders, such as PAD, is linked to OS [52].

PAD patients have increased serum 8-iso-PGF_2α_, and this parameter is an independent predictor for the occurrence of PAD [53], [54]. F2-isoprostane plasma concentrations were, on average, ten times greater in PAD patients than in the control group [55]. These results were observed even in the earliest stages of the disease (Fontaine stages I-II). High levels of serum prostaglandin F2αIII were found by Loffredo et al. [56], [69] in PAD patients at Fontaine stage IIb.

PAD is associated with endothelial dysfunction and systemic atherosclerosis affecting the arterial tree. Previous studies on PAD patients shown improvements in vascular reactivity and walking ability when nitric oxide (NO) generation was restored. According to a prior research by Gresele et al. [57], long-term NO donation reduces the progression of atherosclerosis in PAD patients but fails to improve claudication distance. The goals of pharmacological therapy in PAD are focused on reducing CV risk, preventing critical limb ischaemia (CLI) and improving walking distance.

In patients with CLI, revascularization is the recommended treatment. Iloprost is one of the vasodilator prostaglandins that have been studied as a potentially effective treatment for CLI patients who are not eligible for revascularization surgery. These medications have been given intravenously or intraarterially, either as lengthier infusions (7–28 days) or for shorter periods of time (3–4 days).

Lessiani et al. [58], consistent with a prior publication [59] showed increased OS in CLI patients, as evidenced by urine excretion of 8-iso-PGF_2α_. A third unexpected finding of this study was the considerable decrease in lipid peroxidation observed after one week of iloprost infusion. Patients with higher NO bioavailability following iloprost had substantially lower urine 8-iso-PGF_2α_ levels, as reflected in plasma NOx levels. These findings potentially support the concept that OS in CLI patients is predominantly caused by defective endothelial cells, and that iloprost treatment may reverse, at least in part, lipid peroxidation.

### 4.4 8-iso-PGF_2α_ and Chronic Coronary Syndrome (CCS)

Lipids are the primary target of radical attack in the cardiovascular system. Lipid peroxidation is one important process thought to be responsible for the development of atherosclerosis and blood vessel damage is lipid peroxidation. LDL-C oxidation increases LDL availability for uptake by macrophages, which is a primary factor in the development of plaque, vascular inflammation, and foam cell transformation [60]. IsoP orchestrate a complex inter-relation between OS, platelet activation and smooth muscle cell proliferation, thus mirroring those patients at higher risk for CVD [4].

Strong correlations have been observed between oxidative damage and the initial phases of the atherosclerotic process, indicating the involvement of oxidative compounds in the early stages of atherosclerosis [61]. A research from the Framingham cohort [62] supports the idea that this biomarker may indicate subclinical atherosclerosis and aging by demonstrating a correlation between the urinary excretion of 8-iso-PGF_2α_ and all-cause mortality.

An index of coronary artery atherosclerosis, called coronary artery calcification (CAC), is thought to be a reliable indicator of cardiovascular events [61]. Circulating levels of 8-iso-PGF_2α_ were correlated with CAC in healthy young subjects in the CARDIA Study, independent of C-reactive protein and established CV risk factors, in both genders. The highest frequency of CAC was more prevalent in individuals with high vs low amounts of 8-iso-PGF_2α_ [61].

A study from our group [63] compared urinary 8-iso-PGF_2α_ in 84 patients with heart failure (HF) secondary to CCS, 61 patients with CCS without HF and 42 healthy subjects. Patients with heart failure, not on aspirin, had significantly higher urinary 8-iso-PGF_2α_ than healthy subjects and then CCS patients not on aspirin. Urinary 8-iso-PGF_2α_ excretion, together with plasma NT-pro-BNP, CRP and asymmetric dimethylarginine (ADMA) levels were also significantly higher in the NYHA classes III and IV compared with NYHA classes I and II, both in aspirin-treated and untreated patients.

These results also contribute to suggest a crucial role of OS observed in atherosclerosis onset and progression, especially in the coronary arteries [64].

## 5 8-iso-PGF_2α_ and cardiovascular risk factors

### 5.1 Smoking

Inflammation, OS, and changes in lipid levels are some of the primary processes via which smoking raises the risk of CVD. It is well-established that smoking affects lipid peroxidation and this abnormality is reversible after smoking cessation [65], [66]. A comparison between smokers, previous smokers and non-smokers revealed that smokers tend to have higher levels of 8-iso-PGF_2α_. The meta-analyses by van Der Plas et al. [67] confirmed that urinary 8-iso-PGF_2α_ levels are increased in smokers compared to non-smokers, while further investigations evaluating changes in 8-iso-PGF_2α_, after smoking cessation are needed to evaluate the reversibility of this marker as a clinical risk endpoint.

Healthy smokers had higher urine 8-iso-PGF_2α_ levels compared to age- and sex-matched control participants (Table 1), which also linked with cigarette smoking frequency. Subjects who smoked more than 30 cigarettes per day had greater urine 8-iso-PGF_2α_ levels than those who smoked 15–30 cigarettes per day. Heavy smokers had higher levels of urine cotinine, a persistent metabolite of nicotine, which correlated with urinary 8-iso-PGF_2α_. The particular components of cigarette smoke that contribute to 8-iso-PGF_2α_ excretion in smokers remain unknown. Notably, urinary 8-iso-PGF_2α_ did not drop to levels seen in nonsmokers after quitting smoking. This suggests that quitting smoking for three weeks decreases OS in subjects on long-term, heavy cigarette smoking, but does not completely eradicate it. On the other hand, it's plausible that former smokers were exposed to a high level of passive smoking in their homes or social settings [68].

**Table 1:** Studies considering the urinary excretion of 8-iso-PGF_2α_ in different age-related pathologies, by year of publication and clinical setting.

Authors	Clinical settings	Subjects	Findings
Ciabattoni et al. [167]	Technique validation	Healthy subjects	Development and validation of Radio Immuno Assay for measuring 8-iso-PGF_2α_ in urine samples.
Roberts 2nd et al. [170]	Technique validation	Male volunteers	Development of methods of assay for measurement of urinary 8-iso-PGF_2α_ to obtain an integrated assessment of OS status in vivo in humans over time.
Santilli et al. [63], [103]	Heart Failure	Patients with Heart Failure secondary to ischemic heart disease (IHD) Patients with IHD without Heart Failure Healthy subjects	Patients with heart failure had significantly higher levels of urinary 8-iso-PGF_2α_ as compared with healthy subjects and IHD patients.
Cipollone et al. [46]	Unstable angina	Patients with unstable angina Patients with stable angina Healthy subjects	Patients with unstable angina had considerably higher urinary 8-iso-PGF_2α_ levels compared to stable angina patients and controls.
Rui-Jian Li et al. [171]	Acute coronary syndrome (ACS)	Patients with acute coronary syndrome (ACS) Untreated group Alpha-lipoic acid (α-LA) treatment group	Serum 8-iso-PGF_2α_ levels were significantly lower in the alpha-lipoic acid (α-LA) treatment group than in the untreated group.
Santilli et al. [49]	Acute coronary syndrome (ACS)	Patients with ACS	Higher urinary 8-iso-PGF_2α_ levels were significant predictors of residual, on-aspirin, thromboxane (TX) biosynthesis in ACS.
Lin et al. [172]	Stroke	Patients with acute stroke	Stroke patients showed higher levels of urinary 8-iso-PGF_2α_ compared to controls.
Lessiani et al. [58]	Peripheral artery disease (PAD)	Patients with PAD	Urinary 8-iso-PGF_2α_ levels were dramatically reduced after a daily iloprost infusion for one-week.
Walker et al. [187]	Coronary artery disease (CAD)	Men with stable angina treated with L-arginine or Placebo	Oral L-arginine supplementation reduces the plasma marker of OS 8-iso-PGF_2α._
Gross et al. [186]	Coronary artery disease (CAD)	Young healthy adult men and women	Plasma F(2)-IsoPs were higher in women than in men. F(2)-IsoPs were associated with an increased risk of CAD in both sexes.
Carnevale et al. [188]	Cigarette smoking	Healthy subjects and Smokers	Platelet incubation with 0.1–10 micromolar catechin significantly lowered platelet 8-iso-PGF_2α_ in smokers.
Reilly et al. [68]	Cigarette Smoking	Moderate and heavy smokers	Urinary 8-iso-PGF_2α_ excretion was higher in heavy smokers than moderate smokers and non-smokers. 8-iso-PGF_2α_ urinary levels decreased after cessation of smoking.
Dohi et al. [173]	Hypertension	Patients with hypertension Patients under candesartan treatment Control group	The decrease in C-reactive protein (CRP) levels by candesartan was accompanied by decreases in urinary levels of 8-iso-PGF_2α._
De Faria et al. [69]	Hypertension	Patients with resistant hypertension (RHTN) Patients with well-controlled hypertensive (HT)	Levels of plasma 8-isoprostane were markedly higher in RHTN as compared to HT patients.
Mihalj et al. [70]	Hypertension	Newly discovered patients with essential hypertension treated with -ARB/olmesartan - calcium channel blocker (CCB)/amlodipine plus vitamin C/E or placebo	The magnitude of plasma 8-iso-PGF_2α_ reduction was significantly greater in patients taking vitamins C/E supplementation in the CCB group treatment.
Hirooka et al. [71]	Hypertension	Patients with Hypertension treated with valsartan or amlodipine for one year.	Urinary excretion of 8-iso-PGF_2α_ was significantly reduced in patients treated with valsartan, but not in those treated with amlodipine.
Guagnano et al. [74]	Hypertension	Hypertensive patients with or without microalbuminuria Control subjects	Urinary 8-iso-PGF_2α_ excretion was enhanced in microalbuminuric compared to non-microalbuminuric patients or controls.
Davì et al. [23]	Obesity	Women with android obesity	Women characterized by android obesity had higher levels of urinary 8-iso-PGF_2α_ than nonobese subjects. Successful weight loss was associated with statistically significant reductions in urinary 8-iso-PGF_2α._
Sutherland et al. [174]	Obesity	Overweight subjects	During 6 months of supplementation with vitamin E, plasma 8-isoprostane concentrations decreased significantly (−11%).
Peairs et al. [175]	Obesity	Overweight subjects	Inverse correlation was noted between initial values and changes in several inflammatory and OS urinary marker 8-iso-PGF_2α_ after a short-term low carbohydrate, high-fat weight loss diet.
Loffredo et al. [77]	Obesity	Children with coexistence of hypercholesterolemia and obesity (HOC)	Urinary 8-iso-PGF_2α_ excretion was higher in children with HOC (coexistence of hypercholesterolemia and obesity) compared with healthy subjects.
Vazzana et al. [78]	Obesity	Healthy obese women	Urinary 8-iso-PGF_2α_ and plasma esRAGE were independent predictors of in vivo platelet activation in obese women. A significant decrease in urinary 8-iso-PGF_2α_ was associated with a short-term weight loss program.
Davì et al. [24]	Hypercholesterolemia	Hypercholesterolemic patients Vitamin E supplementation	Urinary 8-iso-PGF_2α_ was significantly higher in patients with hypercholesterolemia than in control subjects. A supplementation of Vitamin E was associated with dose-dependent reductions in urinary 8-iso-PGF_2α_
Puccetti et al. [92]	Hypercholesterolemia	Hypercholesterolemic subjects, previously screened for LOX-1 3′UTR polymorphism, randomized, according to genetic profile (15 T and 15C carriers for each arm) to atorvastatin or rosuvastatin.	After 8 weeks, atorvastatin and rosuvastatin were associated with comparable, significant reductions in urinary 8-iso-PGF_2α_ (39.4% vs. 19.4%).
Juan Ruano et al. [176]	Hypercholesterolemia	Hypercholesterolemic subjects	A meal containing high-phenolic virgin olive oil reduces OS reflected by plasma 8-iso-PGF_2α_ levels.
Anastazia Kei et al. [177]	Hypercholesterolemia	Primary hypercholesterolemia patients Rosuvastatin administration	Plasma F2-isoprostane levels decreased in treated group by 16.7%
Davì et al. [25]	Diabetes	Patients with type 1 diabetes mellitus	Diabetic children showed significantly higher urinary 8-iso-PGF_2α_ levels_._ Statistically significant correlations between IL-6 and urinary 8-iso-PGF_2α_ were observed at diabetes onset.
Santilli et al. [113]	Diabetes	Patients with type 2 diabetes mellitus	A 20-week treatment with acarbose and a 24-week rosiglitazone treatment on top of metformin were associated with significant decrease in serum resistin and urinary 8-iso-PGF_2α_. A direct correlation was observed between resistin and urinary 8-iso-PGF_2α._
Santilli et al. [109]	Diabetes	Patients with type 2 diabetes Healthy subjects	Diabetic individuals had significantly higher urinary 8-iso-PGF_2α_ levels than control patients. There is a noteworthy correlation between the urine excretion rates of 8-iso-PGF_2α_ and 11-dehydro-TXB2 and plasma CD40L in diabetic patients.
Santilli et al. [63], [103]	Diabetes	Newly diagnosed type 2 diabetic patients.	20 weeks of acarbose treatment showed a significantly greater (by 33% vs. baseline) decrease in urinary 8-iso-PGF_2α_ excretion rate. Multiple regression analyses in the acarbose group revealed that mean amplitude of glycemic excursions was the only predictor of 8-iso-PGF_2α_ urinary excretion rate
Mollo et al. [178]	Diabetes	Patients with type 1 diabetes mellitus on α-lipoic acid treatment or placebo	C-reactive protein (CRP) and serum 8-iso-PGF_2α_ levels did not differ between the two groups before and after treatment, showing no changes, compared with pre-treatment values, in either groups.
Liani et al. [110]	Diabetes	Patients with type 2 diabetes mellitus	Urinary 8-iso-PGF_2α_ and diabetes duration independently predicted sCD36 levels.
Ceriello et al. [179]	Diabetes	Patients with type 1 diabetes mellitus	Vitamin C infusion, during induced acute hypoglycaemia, reduces the generation of OS (assessed by plasma 8-iso PGF_2α_) and inflammation.
Tassone et al. [180]	Diabetes	Newly diagnosed type 2 diabetes mellitus patients without any previous clinical evidence of cardiovascular disease	After 4 weeks of aspirin 100 mg/day administration, aspirin significantly increased the plasma and urine 8-iso-PGF_2α_ excretion
Lipsky et al. [189]	Diabetes	Patients with type 1 diabetes mellitus	Children with type 1 diabetes participating in an 18-month behavioral nutrition intervention trial. BMI and body composition indicators were unrelated to urinary 8-iso-PGF_2α_ and adiponectin.
Simeone et al. [79]	Diabetes	Obese patients with prediabetes or newly-diagnosed diabetes Patients treated with Liraglutide Patients on lifestyle changes	At baseline, urinary 8-iso-PGF_2α_ is a significant independent predictor of platelet activation assessed by 11-dehydro-TXB_2_. After achievement of the weight loss target, a comparable reduction in 11-dehydro-TXB_2_ and 8-iso-PGF_2α_ was observed in both arms in parallel.
Li et al. [181]	Diabetes	Patients with type 2 diabetes mellitus Dulaglutide treated group Glimepiride treated group	The amounts of serum 8-iso-PGF_2α_, TNF-α, and IL-6 all decreased significantly in both groups after treatment, and there was no significant difference observable between the two groups.
Santilli et al. [97]	Diabetes	Patients with Impaired glucose tolerance (IGT) Patients with type 2 diabetes mellitus	Urinary 8-iso-PGF_2α_ was comparable in subjects with IGT and diabetes and was significantly higher in subjects with new as compared with established diabetes.
Si Ri Gu Leng Sana et al. [182]	Diabetes	Pregnant women with Diabetes Mellitus (GDM):	Levels serum 8-iso-PGF_2α_ in the natural birth group increased significantly, indicating that epidural analgesia can reduce the inflammatory and OS response of pregnant women with Gestational diabetes mellitus.
Chiarelli et al. [96]	Diabetes	Patients with type 1 diabetes mellitus with early signs of angiopathy Patients with type 1 diabetes mellitus without angiopathy Healthy volunteers	After 6 month treatment with irbesartan, urinary excretion of 8-iso-PGF_2α_ was reduced in cells of adolescents and young adults with diabetic angiopathy.
Pignatelli et al. [183]	Atrial fibrillation	Patients with atrial fibrillation (AF)	In patients with atrial fibrillation urinary 8-iso-PGF_2α_ and NOX2 levels are predictive of cardiovascular events and total mortality.
Molek et al. [184]	Atrial fibrillation	Patients with AF	Increased serum 8-isoprostane levels partly through altered fibrin clot structure are associated with thromboembolic events despite anticoagulant therapy in AF patients.
Vazzana et al. [185]	Chronic kidney disease (CKD)	Patients with CKD (stage 1–4)	Urinary 8-iso-PGF_2α_ was an independent predictor of urinary 11-dehydro-TXB_2_.
Tsuda et al. [190]	Chronic kidney disease (CKD)	Untreated hypertensive subjects with CKD Normotensive subjects	Plasma 8-iso-PGF_2α_ levels were significantly increased in hypertensive subjects in patients with CKD.
Dragani et al. [163]	Hyperhomocysteinemia	MTHFR 677C → T Polymorphism carriers with or without hyperomocysteinemia Non carriers subjects Folic acid supplementation	Urinary 8-iso-PGF_2α_ excretion was higher in carriers with hyperhomocysteinaemia. Folic acid supplementation was associated with decreased urinary 8-iso-PGF_2α_

### 5.2 Hypertension

Increased OS and reduced antioxidative capacity are exhibited by hypertensive patients.

These patients frequently receive angiotensin II (ANGII) receptor type I blockers (ARB) to lower blood pressure (BP), which offer an important counteraction in the maintenance of oxidative homeostasis. Hypertensive patients have higher plasma 8-iso-PGF_2α_ levels, which are related with endothelial dysfunction in resistant hypertension (Table 1) [69]. Recent research examined urine 8-iso-PGF_2α_ levels in individuals using ARB/olmesartan or CCB/amlodipine. Urinary 8-iso-PGF_2α_ levels were favorably correlated with systolic and diastolic blood pressure (BP) in the calcium channel blocker (CCB) (amlodipine) group, but only with diastolic BP levels in patients taking the angiotensin receptor blocker (ARB) olmesartan.

Urinary 8-iso-PGF_2α_ levels were significantly decreased after additional 8 weeks of treatment, independently of the type of antihypertensive therapy; BP levels or absolute levels of 8-iso-PGF_2α_ were not affected by vitamin C/E supplementation, but the magnitude of 8-iso-PGF_2α_ reduction was significantly greater in patients taking vitamin C/E and CCB amlodipine compared to patients receiving only CCB. Furthermore, in the same patients, biomarkers linked with endothelium activation were modulated. Changes in these biomarkers did not show any correlation with the specific action of anti-hypertensive therapy while there was a moderate negative correlation between the endothelial activation marker, sICAM-1 (inflammation indicator), and 8-iso-PGF_2α_ in both groups [70].

In contrast, Hirooka et al. found that the CCB amlodipine was not able to reduce OS while the ARB valsartan lowered urine excretion rate of 8-iso-PGF_2α_ [71].

Mihalj et al. [70] found out a significant negative correlation between aldosterone and 8-iso-PGF_2α_ in the olmesartan group, but not in the amlodipine group. These findings indicated that Angiotensin II has a crucial role in OS in essential hypertension which is unrelated to aldosterone.

Renovascular disease (RVD) is a relatively rare form of secondary hypertension. Renal artery stenosis decreases renal blood flow and perfusion pressure, which frequently activates the renin-angiotensin system. RVD is a prominent cause of end-stage renal failure that is growing more prevalent and is associated with an elevated risk of cardiovascular mortality [72].

Minuz et al. [73] found that hypertensive patients with RVD had significantly higher urine excretion of 8-iso-PGF_2α_ compared to patients with essential hypertension with equivalent blood pressure levels and healthy normotensive participants. In the RVD hypertensive group, urinary excretion of 8-iso-PGF_2α_ was not associated with the presence of conventional cardiovascular risk factors that may independently be associated with increased OS: none of them had hypercholesterolemia, and low HDL cholesterol, diabetes, cigarette smoking, and overt atherosclerosis were equally represented in essential and RVD hypertensives [73].

Microalbuminuria is a predictor of negative outcomes in hypertension. In a recent clinical study, sixty essential hypertensive patients with or without microalbuminuria and 30 controls were studied. Urinary 8-iso-PGF_2α_ was increased in microalbuminuric patients compared to non-microalbuminuric patients or controls [74] (Table 1). Urinary 8-iso-PGF_2α_ excretion rate and microalbuminuria were independently related to 11-dehydro-TXB_2_ in hypertensives, according to a multivariate regression analysis [74].

Thus, while hypertension per se is not associated with enhanced OS, microalbuminuria in hypertensive patients is associated with higher oxidative burden, and increased lipid peroxidation. This evidence is in keeping with the high burden of risk associated with renal involvement, as expression of target organ damage, in cardiovascular risk stratification, according to International Guidelines [75].

### 5.3 Obesity

Obesity is commonly associated with increased OS and low-grade inflammation and both play a crucial role in the development of metabolic and CV disorders [76].

Urinary 8-iso-PGF_2α_ excretion was higher in children with coexistence of hypercholesterolemia and obesity as compared with healthy subject [77] (Table 1).

Our group [23] conducted a cross-sectional comparison of urinary 8-iso-PGF_2α_ excretion levels in 93 women: 44 with a BMI higher than 28 and a waist-to-hip ratio (WHR) of 0.86 or higher (android obese), 25 with a BMI higher than 28 and a WHR lower than 0.86 (gynoid obese), and 24 nonobese women with a BMI lower than 25 (Fig. 2). Multiple regression analysis showed that CRP levels and WHRs of 0.86 or higher predicted the rate of 8-iso-PGF_2α_ excretion, independent of insulin and leptin levels. Out of 20 obese women, 11 successfully lost weight, resulting in substantial decreases in CRP and 8-iso-PGF_2α_ [23].

Several adipocytokines, such as leptin, IL-6, TNF-α, and monocyte chemoattractant protein-1, can be released by visceral fat. These adipocytokines might intensify oxidative events and potentially explain why people with android obesity may have greater levels of OS.

In obese women, a short-term weight loss program increased esRAGE and decreased urine 8-iso-PGF_2α_ and 11-dehydro-TXB_2α_ levels. Multiple linear regression analysis identified urine 8-iso-PGF_2α_ and plasma esRAGE as independent predictors of urinary 11-dehydro-TXB_2_ [78].

Our study found that both lifestyle interventions and liraglutide therapy, a glucagon-like peptide receptor agonist (GLP-1 RA), were equally effective in lowering urine 8-iso-PGF_2α_ levels in obese individuals with prediabetes or newly diagnosed diabetes [79] (Table 1). Weight reduction, independent of therapy, was related with alterations in 8-iso-PGF_2α_ in obese participants. Reductions in urinary 8-iso-PGF_2α_ and TNF-α were shown to be independent predictors of decreases in thromboxane-dependent platelet activation in the examined participants. Thus, weight reduction benefits on thromboxane metabolite excretion, regardless of intervention, may be mediated via a positive influence on lipid peroxidation and inflammation [79].

Mechanisms of enhanced CV risk associated with obesity are partly characterized, and lipid peroxidation may be a mechanistic link between several components of metabolic syndrome and CV diseases, through its role in inflammation [80].

During the first week following Roux-en-Y gastric bypass surgery, the most frequent bariatric operation for patients with extreme obesity, there was a considerable reduction in plasma levels of 8-iso-PGF_2α_. The high preoperative levels (59–199 pg/mL) of plasma 8-iso-PGF_2α_ acutely fell to levels (30–78 pg/mL) that are close to reported values (35 pg/mL) in healthy subjects [81]. Based on these results, it may be concluded that Roux-en-Y gastric bypass helps to restore the redox balance in plasma during the first week following surgery, when significant weight loss is not yet observable but insulin sensitivity has improved [81].

**Fig. 3:**
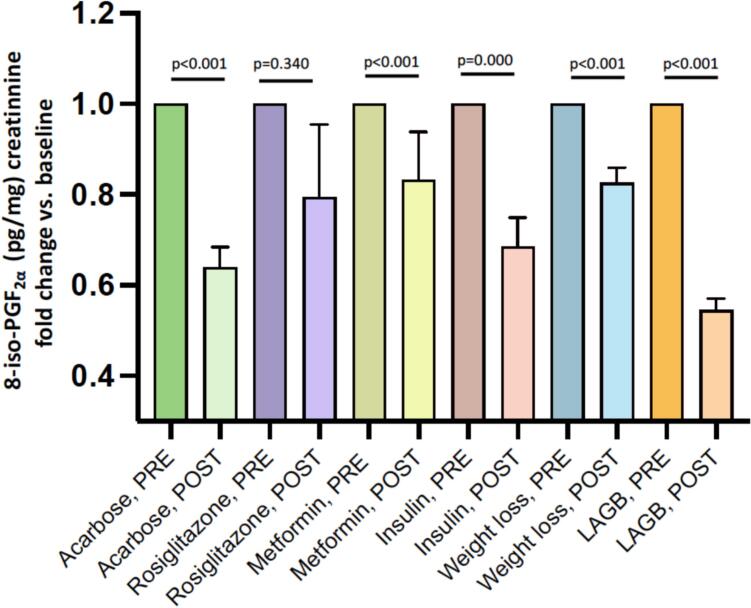
Urinary-8-iso-PGF_2α_ levels in patients with T2DM or obesity, before and after ameliorated metabolic control achieved with antidiabetic therapy or before and after weight loss achieved with lifestyle changes or surgery (laparoscopic adjustable gastric banding). Median (interquartile range) urinary excretion rates of 8-iso-PGF_2α_ have been measured by previously described and validated immunometric method [38]; Urinary 8-iso-PGF_2α_ levels before and after 20 weeks of acarbose treatment in early T2DM patients (*n* = 17), *p* < 0.001 [103]; Urinary 8-iso-PGF_2α_ levels before and after [24 weeks of rosiglitazone treatment in early T2DM patients (*n* = 9), *p* = 0.340 [103]; Urinary 8-iso-PGF_2α_ levels before and after 12 weeks of metformin treatment in newly diagnosed T2DM patients (*n* = 26), p < 0.001 [104]; Urinary 8-iso-PGF_2α_ levels before and after 4 weeks of insulin treatment in T2DM patients (*n* = 21), *p* = 0.0001 [27]; Urinary 8-iso-PGF_2α_ levels before and after weight loss achieved with lifestyle changes or liraglutide treatment in obese patients with prediabetes or early T2DM patients (*n* = 35), *p* = 0.035 [85]; Urinary 8-iso-PGF_2α_ levels before and after 12 months of LABG (laparoscopic adjustable gastric binding) surgery on obese patients (*n* = 30), p < 0.001 [88].

A study from our group [82] that enrolled thirteen obese patients who were scheduled to undergo laparoscopic adjustable gastric banding (LAGB) and evaluated at baseline and after 3, 6, and 12 months, showed that lipid peroxidation assessed by urinary 8-iso-PGF_2α_ progressively and dramatically decreased at any time point following LAGB (Fig. 3).

### 5.4 Hypercholesterolemia

Elevated cholesterol levels are known to play a role in the development of atherosclerotic vascular lesions as well as in cardiovascular events induced by arterial occlusion.

Prior research on increased lipid peroxidation in hypercholesterolemic patients is largely indirect and relied on imprecise measures of lipid oxidation products in plasma or the patient's LDL oxidation susceptibility in vitro [83], [84].

In a study by Davì et al. [26], the majority of patients with hypercholesterolemia had exceptionally increased levels of urinary 8-iso-PGF_2α_ excretion. The association between high LDL-cholesterol levels and abnormal F2-isoprostane generation suggests at least a partial correlation between them [24]. Enhanced biosynthesis of 8-iso-PGF_2α_ may mediate some of the effects of hypercholesterolemia on key factors determining vascular occlusion since this molecule has biological effects on vascular and platelet function [85].

Enhanced formation of 8-iso-PGF_2α,_ was associated with lower bone density and decreased levels of peripheral blood markers of bone formation in hypercholesterolemia [86]. In the study by Mangiafico et al. [86], accordingly with the report by Morrow [87], they did not observe any correlation between lipid parameters and serum 8-iso-PGF_2α_ levels. Conversely, these data differed from those reported by Davì et al. [24] and Reilly et al. [88] on urine samples, with both the latter reporting a positive correlation between the urinary excretion of 8-iso- PGF_2α_ and LDL cholesterol levels. Such a discrepancy may be explained by the measurement of serum levels as opposed to urinary levels, with serum being a less reliable measure, or at least exposed to several pre-analytical issues, as outlined above. According to Davì et al. [24], the majority of patients with hypercholesterolemia had exceptionally increased levels of urinary 8-iso-PGF_2α_ according to a variety of studies, including one by Morrow [87]. The mechanism underlying increased 8-iso-PGF_2α_ in hypercholesterolemia may be more complex than simply explained by higher lipid content or the presence of 8-iso-PGF_2α_ substrate AA. Rather, activation of AA pathway and NAD(*P*)H oxidase pathway may be one crucial event [89]. Platelets generate oxidized LDL (ox-LDL) via NOX2-derived OS. Violi et al. [90] addressed the question whether, once generated by activated platelets, ox-LDL can propagate platelet activation. They discovered that after LDL treatment, washed platelets had higher levels of platelet aggregation and 8-iso- PGF_2α_ generation (+42% and + 53% respectively).

A critical research challenge is to identify new oxLDL mediators. In vascular cells, the main receptor of oxLDL is lectin-like oxLDL receptor-1 (LOX-1), but a putative receptor on T lymphocytes long remained elusive despite a suggested role for oxLDL in adaptive immune responses. The endothelial lectin-like OxLDL receptor-1, or LOX-1, is directly linked to endothelial dysfunction through its involvement in the platelet-endothelium interaction. For platelets, LOX-1 acted as an adhesion molecule. A phosphatidylserine-binding protein called annexin V decreased platelet binding, whereas platelet agonists increased it. Interestingly, platelet attachment to LOX-1 increased endothelin-1 release from endothelial cells, promoting the establishment of endothelial dysfunction and hence advancing the atherogenic process. Through its ability to bind both platelets and OxLDL, LOX-1 may both cause and accelerate atherosclerosis [91].

A further study by our group [92] showed that after 8 weeks, atorvastatin and rosuvastatin were associated with comparable, significant reductions in LDL cholesterol, plasma hs-CRP, urinary TXB_2a_ and 8-iso- PGF_2α_ (Table 1). Patients were previously evaluated for LOX-1 3 UTR polymorphism, according to genetic profile (15 T and 15C carriers for each arm) LOX-1 3′ UTR polymorphism. The impact of rosuvastatin or atorvastatin on CRP, 8-iso-PGF_2α,_ and 11-dehydro-TXB2 did not differ according to the LOX-1 haplotype. On multiple regression analyses, only LDL was a significant predictor of 8-iso-PGF_2α._ In the whole group, combining all the study's data, obtained at baseline and under treatment, 11-dehydroTXB_2_ was significantly correlated with both oxLDL and hs-CRP (all log transformed) and 8-iso-PGF_2α_ was correlated with oxLDL.

Thus, thromboxane-dependent platelet activation, lipid peroxidation, and inflammation are all reduced to a comparable extent by rosuvastatin and atorvastatin. The modifications brought about by either statin do not depend on the existence of the 3′UTR/LOX-1 polymorphism.

### 5.5 Diabetes

Even though DM, especially T2DM, continues to increase around the world, the exact pathogenesis is not perfectly clear. Two pathological processes seem to be involved: inflammation and OS [93].

Indirectly, hyperglycemia can cause ROS generation through the synthesis of AGE and their receptor binding, as well as directly through glucose metabolism and auto-oxidation. NF-kB and PKC are two other signaling molecules that ROS may activate, which may result in the transcription of redox-sensitive genes [94].

OS plays a crucial role in the onset of microvascular and cardiovascular complications associated with diabetes. In patients with T2DM, elevated OS results from metabolic disturbances including hyperglycemia, insulin resistance, hyperinsulinemia, and dyslipidemia. These factors collectively drive excessive mitochondrial superoxide production within endothelial cells.

The central pathophysiological processes underlying this increased OS involve several pathways: (1) enhanced flux through the polyol pathway, (2) elevated generation of advanced glycation end products (AGEs), (3) upregulation of AGE receptors, (4) activation of various protein kinase C isoforms, and (5) heightened activity of the hexosamine biosynthetic pathway [95].

The consequences of OS in T2DM are further exacerbated by the inactivation of endothelial nitric oxide synthase, an essential antiatherosclerotic enzyme.

Early-stage diabetic angiopathy in adolescents and young adults with type 1 diabetes mellitus (T1DM) is characterized by abnormal intracellular antioxidant enzyme synthesis and activity, which may be ameliorated by angiotensin-receptor blocker therapy (Table 1) [96].

Since the early nineties, our group demonstrated enhanced TX biosynthesis and lipid peroxidation in T2DM, directly related to markers of glycemic control such as fasting plasma glucose and HbA1c, and that a tight metabolic control can affect the rate of thromboxane formation and lipid peroxidation in vivo [26].

In poorly controlled diabetes, reduced plasma antioxidant capacity and increased lipid hydroperoxides and 8-iso-PGF_2α_ were appreciated; conversely, improved metabolic control and vitamin E supplementation were associated with significant reduction in both urinary 8-isoPGF_2a_ and 11-dehydro-TXB_2_ [26].

In children and adolescents with T1DM, increased lipid peroxidation has been observed at disease onset, alongside heightened thromboxane-mediated platelet activation, both likely linked to an acute inflammatory response. Notably, significant correlations were found between interleukin-6 (IL-6) and levels of both 8-iso-PGF_2α_ and 11-dehydro-TXB_2_. When these patients were reassessed after one year—once the inflammatory phase had subsided—they exhibited a marked decrease in lipid peroxidation and platelet activation, corresponding with lowered IL-6 and tumor necrosis factor-alpha (TNF-α) levels [25].

Building on this, we investigated whether similar biochemical changes occur in long-standing T2DM or are already present during the early stages of the disease. By comparing F2-isoprostane production and thromboxane-dependent platelet activation among individuals with impaired glucose tolerance (IGT) and T2DM patients diagnosed either less than 12 months or more than 12 months prior, we found that urinary excretion of 11-dehydro-TXB2 and 8-iso-PGF_2α_ at baseline was similarly elevated across all groups, with minimal variability within subjects over time [97].

A more recent trial from our group confirmed that obese patients with prediabetes or newly-diagnosed T2DM show higher rates of platelet activation and lipid peroxidation [79], and obtained a significant reduction in levels of 11-dehydro-TXB_2_ and 8-iso-PGF_2α_ after successful weight loss, achieved with lifestyle changes or an incretin-based therapy with liraglutide (Fig. 3).

The above-mentioned body of evidence led to the idea that, in addition to chronic hyperglycemia, glycemic instability with normal or near normal fasting plasma glucose but prevalent postprandial hyperglycemia, may be responsible for enhanced lipid peroxidation in the early and possibly preclinical stages of diabetes.

Postprandial glucose (PPG) is a marker of poor diabetes management and represents the shift from prediabetes to overt diabetes. Postprandial hyperglycemia is an independent risk factor for diabetes-associated morbidity and death. OS is one of the best characterized mechanisms by which glucose fluctuations during the postprandial period exert their deleterious effects, contributing to platelet activation [98], [99], [100].

Monnier et al. demonstrated that glucose fluctuations showed a triggering effect on OS in diabetic patients [100].

The duration and extent of chronic persistent hyperglycemia and the acute variations in glucose, around a mean value over a daily period, are two factors that determine exposure to glycemic disorders [101].

Urinary 8-iso-PGF_2α_ excretion rates were shown to be substantially linked with acute glucose swings [102]. However, no correlation was seen when plotted against key indicators of chronic hyperglycemia (HbA1c and mean daily glucose concentrations).

We found increased lipid peroxidation in response to postprandial hyperglycemic spikes in early T2DM patients with HbA1c levels, no evident microvascular or macrovascular complications, and prolonged TX-dependent platelet activation in vivo [103].

Time-dependent downregulation of urinary TX metabolite excretion and 8-iso-PGF_2α_ was produced by a moderate decrease in PPG obtained with acarbose administration. This suggests that early metabolic issues, platelet activation, and lipid peroxidation are causally related in this setting [98].

The effect of glycaemic variability on OS and endothelial function in healthy subjects and T2DM patients was examined with a euinsulinemic hyperglycemic clamp, to compare three different glycemic profiles over 24 h: (1) 10 mmol/L (180 mg/dL) persistently, (2) 15 mmol/L (270 mg/dL) persistently, and (3) 5 and 15 mmol/L (90 and 270 mg/dL) every 6 h.

Glycaemic variability produced greater endothelial dysfunction and OS, assessed by plasma 3-nitrotyrosine and 24-hour urinary excretion rate of 8-iso-PGF_2α_ compared with persistent, either 10 or 15 mmol/L (180 or 270 mg/dL) glucose [104].

More recently, continuous glucose monitoring (CGM) has become a valid and reliable tool for measuring glucose fluctuations [105].

In a study by Dimova et al. [106] OS markers were positively correlated with postload plasma glucose in patients with prediabetes even after adjustment for the presence of hypertension and smoking. In another study [107] Liraglutide (a GLP-1 receptor agonist) add-on therapy to continuous subcutaneous insulin infusion (CSII), not only led to ameliorated glycemic control but also lowered the glycemic variability and OS in poorly controlled newly diagnosed T2DM.

Blood and urine samples were obtained at 24 and 72 h, and the Continuous Glucose Monitoring System (CGMS) was used to assess the glucose profiles throughout an interval of 72 h. Metformin combined with insulin analog therapy greatly decreased glucose fluctuations. Following insulin analog plus metformin therapy, there was a significant reduction in plasma and urine lipid peroxidation [108].

Glucose fluctuations, typical of prediabetes but also frequent in poorly controlled overt diabetes, foster lipid peroxidation and platelet activation.

**Fig. 4:**
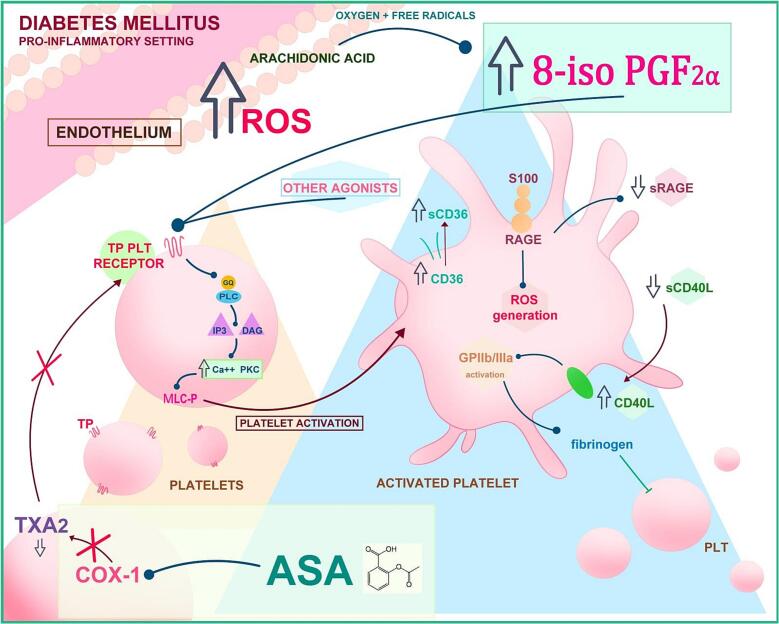
Role of lipid peroxidation on platelets in the pathophysiology of atherothrombosis in DM. Pro-inflammatory conditions in DM lead to the accumulation of ROS that in the presence of arachidonic acid generates lipid peroxidation and increased levels of 8-iso-PGF_2α_. Activated platelets can further increase OS conditions by the release of molecules such as CD40L, sCD36 and sRAGE. ASA acts as an acetylating agent, inducing the acetylation of COX-1 thus consequently inhibiting TXA2 synthesis and its role in the activation of TP receptor in platelets. (Abb. Acetyl Salicylic Acid, ASA; Cycloxigenase 1, COX-1; Thromboxane A2, TXA_2_; Platelets, PLT; Myosin Light Chain, MLC; Protein Kinase C, PKC; Inositol trisphosphate, IP3; thromboxane receptor, TP; diacylglycerol, DAG; Reactive oxygen species, ROS).

Inflammatory mediators derived from platelets expand the functional repertoire of platelets from players of hemostasis and thrombosis to powerful amplifiers of inflammation (Fig. 4).

It is shown that a number of inflammatory reactions in atherosclerosis are mediated by CD40 signaling. Many different types of inflammatory cells express CD40 ligand (CD40L), and endothelial cells, that are stimulated by pro-inflammatory cytokines, are also able to express CD40L. However, soluble CD40L (sCD40L) is primary derived from platelets (>95%).

Elevated soluble CD40L levels have been observed in both type 1 and 2 diabetes. Diabetics had considerably greater levels of 8-iso-PGF_2α_, 11-dehydro-TXB_2_, sCD40L, and CRP than control individuals. Multiple regression analysis showed that 11-dehydro-TXB_2_ and 8-iso-PGF_2α_ excretion rates predicted sCD40L levels [109].

A 88 kDa glycosylated transmembrane protein, named CD36 is expressed by a variety of cell types, including endothelial and macrophage cells.

Ox-LDL activates platelets through a unique CD36-dependent signaling mechanism. A cross-sectional study compared 200 diabetes patients and 47 healthy people for platelet-mediated inflammatory markers (sCD36, sCD40L), urine 11-dehydro-TXB_2_, and 8-iso-PGF_2α_, and *in vivo* indicators of platelet activation and lipid peroxidation. sCD36 showed a linear correlation with sCD40L. Multiple regression study identified 11-dehydro-TXB_2_, 8-iso-PGF_2α_, and diabetes duration as independent predictors of sCD36 levels [110].

RAGE has been implicated in obesity-related metabolic conditions and accelerated atherothrombosis.

Soluble (s) RAGE isoforms, including the endogenous secretory splice variant (esRAGE), are detectable in plasma and various tissues. Acting as decoy receptors, sRAGE molecules neutralize the effects of advanced glycation end-products (AGEs), potentially mitigating inflammatory responses triggered by RAGE ligands. Several clinical studies suggest that levels of sRAGE and esRAGE may serve as important markers of RAGE activity in diabetes. This information could prove valuable for assessing vascular risk in affected patients.

Low sRAGE levels are also associated with enhanced in vivo OS and endothelial dysfunction in T2DM patient, as reflected by urinary 8-iso-PGF_2α_ excretion and plasma asymmetric dimethyl-arginine (ADMA), respectively [111].

These findings provide support to the remarkable hypothesis that ligand-RAGE hyperactivity, associated with poor glycemic control, increases in vivo ROS production, which in turn promotes endothelial dysfunction, platelet activation, and systemic inflammation.

Resistin is an adipokine known to promote inflammation and insulin resistance by affecting various cell types, including platelets. It belongs to a family of cysteine-rich secretory proteins produced during the breakdown of white adipocytes and is implicated in the development of insulin resistance and glucose intolerance [112].

Our research group [113] found that a 20-week treatment with acarbose, an α-glucosidase inhibitor, in patients with T2DM led to significant reductions in serum resistin and 8-iso-PGF_2α_ levels, with a direct correlation observed between changes in serum resistin and urinary 8-iso-PGF_2α._ Moreover, adding a 24-week rosiglitazone treatment, a PPARγ agonist, to metformin therapy resulted in significant decreases in resistin and 8-iso-PGF_2α_ levels, alongside a reduction in HOMA index (Fig. 3). These findings suggest that resistin may contribute to insulin resistance and oxidative stress by partially antagonizing insulin action through PPARγ pathways.

Overall, low grade inflammation triggered by hyperglycaemia may elicit lipid peroxidation that contributes to TX-dependent platelet activation. Activated platelets, in turn, may amplify OS and platelet activation through the release of molecules such as CD40L, sCD36, es RAGE, and resistin (Fig. 4).

### 5.6 Chronic kidney disease (CKD)

Chronic kidney disease (CKD) affects more than 10% of the adult population globally.

Moderate and severe chronic kidney disease (CKD) are classified as having a high and very-high CVD risk status according to the 2021 European Society of Cardiology (ESC) guidelines [114] on cardiovascular disease (CVD) prevention, despite other variables like age. However, estimated glomerular filtration rate (eGFR) and albuminuria were not included in its algorithms to predict CVD risk for apparently healthy subjects, systemic coronary risk estimation 2 (SCORE2) and systemic coronary risk estimation 2 in older persons (SCORE2-OP).

OS has been proposed as a significant risk factor for CVD in people with CKD, resulting from an imbalance between decreased antioxidant defences and increased of free radicals generation caused by malfunctioning mitochondria [115].

The majority of the data supporting increased lipid peroxidation in CKD is indirect since it is based on crude measures of lipid oxidation products in plasma [116].

Plasma 8-iso-PGF_2α_ levels were significantly higher in patients under hemodialysis (HD) and continuous ambulatory peritoneal dialysis (CAPD) patients (346.3 ± 132.4 pg/mL; range 49.8–870) than in age-matched control subjects (150.9 ± 61.6 pg/mL; range 33.5–235). In addition, 8-iso-PGF_2α_ concentration was significantly higher in HD patients (389.8 ± 148.3 pg/mL) than in CAPD patients (254.3 ± 76.6 pg/mL). Plasma 8-iso-PGF_2α_ concentration was linearly correlated with serum haptoglobin and CRP [117].

These data raise the hypothesis of a crucial molecular relation between inflammation, increased atherosclerosis, and lipid peroxidation in the uremic environment. Even in the pre-uremic setting, we showed that compared to stage 1–2 subjects, patients in stage 3–4 CKD exhibited enhanced lipid peroxidation, as reflected by increased urinary 8-iso-PGF_2α_ levels. As the degree of renal failure worsened, lipid peroxidation gradually increased. The levels of F2-isoprostane and GFR were similarly highly linked [118].

As already mentioned, 8-iso-PGF_2α_ acts as an agonist on platelet aggregation via Thromboxane A2 receptor (TXA2-R). Intra-arterial infusion of F2-IsoPs led to a reduction in GFR and these effects were counteracted with TXA2-R antagonists [119].

When considered together, these results imply that F2-Isoprostane production might contribute to the occurrence and worsening of CKD.

### 5.7 Combined cardiovascular risk factors and synergistic effects on oxidative stress

In clinical practice, CV risk factors seldom occur in isolation but rather tend to cluster within the same individual, particularly in the context of metabolic syndrome and T2DM. This coexistence is not merely additive, but may result in synergistic or even multiplicative effects on oxidative stress, thereby amplifying lipid peroxidation and its downstream consequences.

Several of these conditions share common and interrelated pathophysiological mechanisms. Chronic low-grade inflammation, a hallmark of obesity and insulin resistance, promotes the activation of enzymatic sources of reactive oxygen species, including NADPH oxidase (notably NOX2), as well as mitochondrial dysfunction. In parallel, hyperglycemia and glycemic variability further enhance oxidative stress through multiple biochemical pathways, including advanced glycation end-product (AGE) formation, protein kinase C activation, and increased flux through the polyol and hexosamine pathways.

Dyslipidemia, particularly elevated levels of oxidized LDL, may further propagate oxidative damage and platelet activation, while hypertension contributes to endothelial dysfunction and impaired nitric oxide bioavailability. The convergence of these mechanisms results in a self-perpetuating cycle of oxidative stress, inflammation, and vascular dysfunction, which may substantially increase the formation of F2-isoprostanes such as 8-isoPGF_₂α_.

Importantly, experimental and clinical evidence suggests that 8-iso-PGF_₂α_ not only reflects oxidative stress but may also actively contribute to platelet activation and thrombus formation, particularly in the presence of subthreshold concentrations of other agonists. Therefore, the coexistence of multiple CV risk factors may enhance both the production and the biological effects of 8-isoPGF_₂α_, ultimately leading to a higher thrombotic risk.

Despite strong mechanistic plausibility, studies specifically designed to quantify the combined impact of multiple coexisting risk factors on 8-iso-PGF_₂α_ levels are still scarce. Future research should aim to address this gap by adopting integrated and multidimensional approaches, which may improve CV risk stratification and help identify patients who could benefit from targeted therapeutic interventions aimed at reducing oxidative stress.

## 6 Role of 8-iso-PGF_2α_ as marker of aspirin insensitive thromboxane biosynthesis: implications for aspirin response

As with any other antithrombotic therapy, patients taking acetylsalicylic acid may experience recurrent events (treatment failure) due to the multifactorial nature of atherothrombosis. This phenomenon was characterized by evidence of less-than-expected inhibition of platelet function [120].

This concept was later renamed as “interindividual variability” in the response to acetylsalicylic acid [121].

The definition “suboptimal aspirin response” does not differentiate between suboptimal effect as an inhibitor of COX-1 and TXB2 formation, which has therapeutic implications in terms of aspirin dosing regimen, and failure of aspirin to inhibit the aggregation response (this may result from either inherent platelet hyperactivity or a failure to suppress COX-1), which has been associated with poor clinical outcomes.

A large body of evidence supports the contention that OS, may be responsible, at least in part, for less-than-expected response to aspirin. A pathophysiological mechanism related to OS and contributing to suboptimal aspirin action or responsiveness includes F2-isoPs, acting as partial agonists of thromboxane receptor (Fig. 4).

Moreover, by enhancing the platelet response to common agonists through glycoprotein (Gp) IIb/IIIa activation, IsoPs could play a role in the propagation of platelet activation [122].

As already stated, F2-isoPs are chemically stable compounds that can partially activate the thromboxane receptor (TP) in a COX- independent manner, in the presence of subthreshold concentrations of other agonists [123].

As a result, an inappropriate isoPs synthesis might prevent aspirin's inhibitory effects. Platelet TP receptor agonists, that are not responsive to aspirin, such as and TXA2 produced from COX-2, and F2-isoPs may be responsible for the incomplete inactivation of platelets observed in T2DM. Blocking the interaction of both aspirin-sensitive and aspirin-insensitive agonists with this platelet receptor should theoretically provide a wider protection against the anticipated detrimental effects of platelet activation.

Against the expectations, the PERFORM trial, of terutroban (a TP-receptor agonist) versus aspirin in 18,000 stroke patients was halted on the basis of an interim analysis failing to support the superiority hypothesis.

In contrast to the theory that aspirin nonresponsiveness may be attributed to isoPs production, in subjects with stable CAD and on daily aspirin therapy, inadequate platelet response to aspirin, defined as residual platelet aggregation >20% per AA-induced light transmission aggregometry, was not associated with higher urinary excretion of 8-iso-PGF_2α_ [124]. However, aspirin response has been evaluated with a functional method that does not reflect the mechanism of action of the drug, and that is characterized by poor intrasubject coefficient of variation. Moreover, an arbitrary threshold has been chosen to assess resistance.

ROS are deeply linked to platelet cell activation. When platelets are activated by common agonists, they release various ROS, such as hydrogen peroxide or superoxide anion, which perpetuate platelet aggregation [125].

Among the various enzymatic pathways involved in the generation of reactive oxygen species (ROS) and the subsequent formation of intraplatelet 8-iso-PGF_2α_, NOX2—the catalytic component of the NADPH oxidase complex—emerges as a key contributor. This is evidenced by the marked reduction in both urinary and platelet levels of 8-iso-PGF_2α_ observed in individuals with a hereditary deficiency of NOX2 [126].

Platelets themselves are capable of producing ROS, with NADPH oxidase being the primary enzymatic source. In platelets, ROS play several roles: (a) they amplify platelet activation, (b) facilitate the release of platelet agonists such as ADP, promoting the formation of isoprostanes and oxidized LDL (ox-LDL), and (c) contribute to the release of pro-atherogenic mediators like CD40 ligand (CD40L). Platelets express all components of the NADPH oxidase complex, including gp91^phox^ (also known as NOX2), the enzyme's catalytic core [51].

When platelets from healthy patients were incubated with a high concentration of 8-iso-PGF_2α_, there was a significant increase in platelet recruitment. Reduced 8-iso-PGF_2α_ synthesis was observed in platelets lacking NADPH oxidase, concurrent with decreased platelet recruitment.

Indirect evidence of ROS as triggers of platelet activation also derives from by experiments using animal knock-outs for antioxidant enzymes [127] or pharmacologic tools to inhibit NADPH oxidase-dependent ROS formation and by clinical studies with antioxidants such as statins.

Statins reduce platelet ROS generation, through a mechanism involving NADPH oxidase down-regulation.

A distinctive feature of statins lies in their capacity to suppress the formation of both thromboxane A2 (TXA2) and 8-iso-PGF_2α_ in platelets by inhibiting NOX2 activation. In a study by Pignatelli et al., administration of atorvastatin (40 mg) to patients with hypercholesterolemia resulted in a rapid reduction of NOX2 activity, accompanied by a decrease in platelet isoprostane levels [128].

Atorvastatin may exert a direct modulatory effect on platelet function. In vitro experiments have demonstrated a dose-dependent (0.1–10 mM) inhibition of NOX2-driven oxidative stress by atorvastatin, which in turn leads to a reduction in 8-iso-PGF_2α_ production and phospholipase A2 (PLA2) activation—ultimately decreasing TXA2 synthesis by platelets [129].

Furthermore, acute treatment with rosuvastatin (20 mg) in individuals with hypercholesterolemia has been shown to significantly reduce the release of soluble NOX2-derived peptide (sNOX2dp), a specific biomarker of NADPH oxidase activity [33].

By reducing platelet ROS generation, several disorders worsened by atherothrombosis may benefit from statins antiplatelet and antioxidant activities.

Low-dose acetylsalicylic acid may inhibit COX-1 in DM, leading to a rise in oxidant species-mediated conversion of AA to isoPs in platelets. This is connected with NOX2 activation and increased platelet recruitment. Increased platelet isoPs production would counteract TXA2 inhibition, reducing the antiplatelet action of acetylsalicylic acid [130].

Aspirin had no effect on platelet isoP synthesis in non-diabetic individuals, but it increased intraplatelet isoPs in DM patients 3 and 7 days after starting aspirin treatment, which correlated with increased platelet ROS formation and NOX2 activation, implying a cause-and-effect relationship between NOX2-generated ROS and platelet isoPs over-generation [131].

Ex vivo studies utilizing a particular NOX2 inhibitor to incubate platelets corroborate this theory.

## 7 Antioxidant supplementation

Supplementation with antioxidants represents a functional strategy to support oxidative defense, but the evaluation of its effectiveness inevitably requires a marker which is able to verify the impact of such supplementation on the oxidative system.

8-iso-PGF_2α_ has been considered one of the most reliable markers of OS [132] and it's concentration in plasma or urine has been used to evaluate the influence of antioxidants/nutraceutical products administration in several studies.

Among the best-known antioxidants, vitamin C, also known as ascorbic acid, occupies a role of major importance. Ascorbic acid is an essential micronutrient, which cannot be synthesized by the human body [133] and which has got a well-established antioxidant activity playing a considerable role in aging and age-related disorders [133].

In 2010 Basili et al. evaluated whether ascorbic acid infusion was able to affect microcirculatory perfusion in patients undergoing elective percutaneous coronary intervention (PCI) for stable angina [134]. Previous studies observed an increase in both OS and 8-iso-PGF_2α_ values post-PCI, predisposing patients to a higher risk of the onset of impaired periprocedural perfusion [88], [135]. Basili et al. demonstrated not only an improvement in microcirculatory perfusion in patients treated with vitamin C compared to patients treated with placebo, but also a significant reduction in serum 8-iso-PGF_2α_ levels corroborating the hypothesis of the involvement of OS in the development of post-procedural perfusion impairment. In addition, a significant increase in 8-iso-PGF_2α_ levels has been observed in placebo-treated patients in agreement with previous studies results [134].

Another study provided evidence for the antioxidant role of ascorbic acid and its ability to modulate the expression of 8-iso-PGF_2α_: starting from the observation that cigarette smoke contains a large amount of reactive oxygen species [136] and taking into account that in non-smokers subjected to second hand smoke there are reduced plasma levels of antioxidants [137] which is indicative for an involvement of antioxidants in counteracting the effects of such exposure, Dietrich et al. designed a randomized, double-blind and controlled study aimed at evaluating the expression of OS in non-smoking patients exposed to second hand smoke and subjected to supplementation with Vitamin C, a blend of antioxidants or placebo.

The result of this study highlighted a significant reduction in plasma levels of 8-iso-PGF_2α_ in patients treated with ascorbic acid alone and with the mixture of antioxidants compared to the control group treated with placebo [137].

In addition to ascorbic acid, vitamin E and Beta carotene have also demonstrated antioxidant properties [138]. A previous study by G. Davì et al. demonstrated a significant reduction in urinary 8-iso-PGF_2α_ levels following supplementation with Vitamin E in patients with diabetes mellitus [19]. The same result was obtained by the study by Guertin K.A. et al. who evaluated whether a long-term supplementation with vitamin E and/or selenium was able to reduce OS in smokers using urinary 8-iso-PGF_2α_ as a reference marker. The study demonstrated a significant reduction in urinary 8-iso-PGF_2α_ levels in subjects treated with vitamin E compared with placebo and it was also observed that the intake of selenium in combination with vitamin E did not determine additional effects on 8-iso-PGF_2α_ levels while taking selenium alone resulted in no significant reductions in 8-iso-PGF_2α_ compared to placebo [139].

In healthy patients with low grade OS, therapeutic doses of vitamin E supplementation had no effect on lipid peroxidation and thromboxane production in vivo. These findings support the theory that one of the main factors, influencing the antioxidant effect of vitamin E administration, is the basal rate of lipid peroxidation [140].

However, the lack of evidence of a significant effect on urinary 8-iso-PGF_2α_ levels clashes with the results of other studies which recognize antioxidant properties in selenium. Indeed, selenium has been shown not only to be involved in metabolic homeostasis and in the correct functioning of the immune system, but also to have an important role in aging and consequently in the oxidative defense [141]. Selenium deficiency is also associated with the onset of cardiovascular disease, infertility and it also seems to be associated with cognitive decline, all conditions strictly related to the OS and aging [142]. Actually, some studies have demonstrated a correlation between serum selenium concentration and 8-iso-PGF_2α_ levels both in rats subjected to diets low in selenium [143] and in humans where higher serum selenium levels are associated with reduced levels of urinary 8 -isoPGF_2α_ [144].

About vitamin D, heterogeneous result have been shown in the major trials aimed at evaluating its antioxidant effect in healthy subjects [145], [146] as well as in diabetic subjects there was no clear antioxidant effect [147]. Heterogeneous results have also been obtained in studies aimed at evaluating the effect of vitamin D supplementation on the expression of 8-iso-PGF_2α/_F2-IsoPs levels [148].

Polyphenols also constitute a group of natural substances with known antioxidant effects. Polyphenols commonly included in our diet are flavanols, flavanones, hydroxycinnamates, flavonols and anthocyanins [149], abundantly present in vegetables, fruits and beverages derived from them. Due to their diffusion and the impact, they have on the diet, some studies aimed at evaluating whether the antioxidant action of these molecules also acts on the expression of 8 isoPGF_2α_ levels when used as a supplement. A 2011 randomized, double-blind, placebo-controlled, study conducted by B. V. Nemzer et al. on 31 healthy patients observed that the single intake of a drink rich in polyphenols led to a reduction in both the levels of advanced oxidation protein products (AOPP) and serum 8-iso-PGF_2α_ levels by 39% and 40%, respectively [150].

Similar results have been obtained from studies which have evaluated the effects of eating foods rich in polyphenols, such as for example cocoa.

Cocoa, which is a food of considerable interest in the nutraceutical field thanks to its high amount of flavonoids, has shown both antioxidant properties and the ability to reduce inflammation [151]. Furthermore it seems to be able to delay cellular damage by acting on the formation of free radicals and also showed a protective action against some pathologies such as coronary heart disease, cancer and neurodegenerative disease [152].

A recent systematic review and meta-analysis of interventional studies has shown that cocoa significantly reduces the expression of 8-iso-PGF_2α_ as well as malondialdehyde, which is also a marker of OS, without affecting the expression levels of the other studied markers such as ferric-reducing antioxidant potential (FRAP), total radical-reducing antioxidant potential (TRAP) and oxidized low-density lipoprotein [153].

It is still unclear how polyphenols impact on OS, the benefits brought by these molecules have been repeatedly observed but their relationship between supplementation and antioxidant activity is not in agreement with all studies, since even the dose seems to have a crucial relevance in determining the antioxidant effect. Indeed D. Mastroiacovo et al. demonstrated how the constant intake of Cocoa flavonoids is able to bring cognitive benefits mainly when the supplementation occurs at high or intermediate concentration and to a lesser extent at low concentrations even if the evidence of improvement persists in both situations. Considering flavonoids antioxidant function, these benefits should be mainly attributed to an effect on OS. Consistently, the supplementation with high and intermediate concentrations of cocoa flavonoids determines a significant reduction in 8-iso-PGF_2α_ levels (respectively *p* = 0.002 and 0.0065) but this significance is lost at low concentrations [154]. Furthermore, even dark chocolate, just like cocoa, has got flavonoids in large quantities, however studies come to conflicting conclusions about its effect on 8-iso-PGF_2α_ levels [155], [156].

Polyunsaturated fatty acids also have demonstrated significant beneficial properties, especially in the cardiovascular field [157]. In particular, omega-3 fatty acids seem to perform various cardioprotective functions, indeed a diet rich in omega-3 fatty acids is associated with lower serum cholesterol, triglycerides and lower blood pressure as well as stabilization of atherosclerotic plaques [158]. Their anti-inflammatory and immunoregulatory action are also known [159]. Among the multiple effects determined by the omega-3 fatty acids, the role they play in OS is also of considerable importance since it is known that these molecules can act as enhancing agents for the oxidative defense against ROS [160]. Even in this case, some studies investigated on the correlation between supplementation with omega-3 fatty acids and 8-iso-PGF_2α_ levels. In a 2006 study Nälsén C. et al. demonstrated that the supplementation of omega-3 fatty acids in 162 healthy subjects is able to reduce the serum levels of 8-iso-PGF_2α_ [161]. This result is consistent with the data obtained from subsequent studies [162].

Our group showed that subjects with the MTHFR 677C → T polymorphism and moderate hyperhomocysteinemia have increased production of 8-iso-PGF_2α_ which causes persistent platelet activation in this scenario [163].

Patients with hyperhomocysteinemia who carry the minor allele of the MTHFR gene experienced a more pronounced reduction in total homocysteine levels, along with decreased lipid peroxidation and platelet activation, following folic acid supplementation compared to hyperhomocysteinemic individuals without this allele [163] (Table 1, Fig. 2).

Several pharmacological and lifestyle interventions have been shown to reduce 8-iso-PGF₂α levels, including statins, antioxidant supplementation, glucose-lowering therapies, and weight loss strategies. However, the relationship between treatment intensity and the magnitude of reduction in 8-iso-PGF₂α remains insufficiently characterized.

Available evidence suggests that more intensive therapeutic interventions may be associated with greater reductions in oxidative stress markers. For instance, higher doses of statins or more effective lipid-lowering strategies have been linked to larger decreases in lipid peroxidation, likely through both lipid-lowering and pleiotropic anti-inflammatory effects. Similarly, antioxidant supplementation (e.g., vitamin E) has demonstrated dose-dependent effects in some studies, although results are not entirely consistent.

In the context of metabolic interventions, improvements in glycemic control and reductions in glycemic variability appear to correlate with decreases in 8-iso-PGF₂α levels, suggesting a potential gradient effect related to metabolic stabilization. However, these observations are often derived from heterogeneous study designs and indirect comparisons, rather than from studies specifically designed to assess dose–response relationships.

Overall, the lack of standardized, dose-gradient studies limits the ability to define precise thresholds of therapeutic efficacy in terms of oxidative stress reduction. This represents a critical gap in the current literature and highlights the need for well-designed prospective studies aimed at establishing quantitative relationships between treatment intensity and changes in 8-iso-PGF₂α. Such data could enhance the clinical applicability of this biomarker, particularly in guiding personalized therapeutic strategies.

## 8 Conclusions

Several cardiovascular conditions are characterized by elevated isoPs generation and excretion. Moreover, isoPs are involved in the pathophysiology of CVD by activating the TP receptor. While a contributory role of 8 isoPGF_2α_ in CVD has been suggested, measurement of isoPs concentrations in urine, plasma or further body fluids may represent a potentially valuable biomarker reflecting oxidative stress status and its association with CV complications, rather than establishing a direct causal or predictive role. Indeed, formation of isoPs may be suppressed by several therapeutic strategies targeting OS or the initial metabolic abnormalities triggering ROS formation and lipid peroxidation. Formation of F2-IsoPs, with particular reference to 8-iso-PGF_2α_, is an important initiator and booster for platelet activation, with a mechanism not inhibited by antiplatelet agents, namely aspirin, due to the agonism of TP receptor.

Consequently, pathways involving isoPs may partly explain the reduced efficacy of low-dose aspirin observed in certain clinical settings. Further research is required to better clarify these associations, and to explore alternative antithrombotic strategies targeting oxidative stress (OS).

Combining statins with aspirin may provide additional benefits, although definitive conclusions require longitudinal and interventional data.

Future studies should aim to clarify whether specific patient subgroups—possibly characterized by elevated 8-iso-PGF_2α_ levels—might derive benefit from targeted therapeutic approaches; however, such hypotheses require confirmation in adequately powered prospective investigations.

Potential limitations of measuring F2-IsoPs is that they can be generated ex vivo in biological fluids such as plasma in which arachidonyl-containing lipids are present, and the paucity of large scale, longitudinal study that evaluate, in patients with CVD, the prognostic role of 8 isoPGF_2α._ Thus, further population-based studies are needed.

In conclusion, lipid peroxidation is a pathophysiological hallmark shared by cardiovascular, neoplastic [164] and neurodegenerative diseases [165], [166].

A comprehensive analysis into each disorder is crucial for researchers to better understand how lipid peroxidation could play a role in each disease's pathogenesis.

Consistently, detection of 8-iso-PGF_2α_ in apparently healthy subjects may reflect increased oxidative stress burden; however, its role as a predictive biomarker for future disease development remains to be established. Integration with traditional and non-traditional risk factors is essential, and conclusions regarding risk stratification should be drawn cautiously. Given the cross-sectional nature of most available data, current evidence primarily supports associations rather than causal or predictive relationships.

## CRediT authorship contribution statement

**Paola Simeone:** Writing – review & editing, Writing – original draft, Conceptualization. **Rossella Liani:** Writing – original draft, Formal analysis, Data curation, Conceptualization. **Stefano Lattanzio:** Writing – review & editing, Data curation. **Maurizio Frezza:** Writing – original draft. **Margherita Alfonsetti:** Writing – review & editing, Writing – original draft. **Francesco Cipollone:** Writing – review & editing. **Francesca Santilli:** Writing – review & editing, Writing – original draft, Funding acquisition, Data curation, Conceptualization.

## Declaration of Generative AI and AI-assisted technologies in the writing process

During the preparation of this work, the authors used ChatGPT4 developed by OpenAI, in order to improve language and grammar. After using this tool, the authors reviewed and edited the content as needed and take full responsibility for the content of the publication.

## Funding

This work was supported by the 10.13039/501100000780European Union-Next Generation EU Under the Italian Ministry of University and Research (MUR) National Innovation Ecosystem Grant ECS00000041-VITALITY-D73C22000840006 Recipient: FS Fondo per il Programma Nazionale di Ricerca e Progetti di Rilevante Interesse Nazionale (PRIN) 2022ZE9E4Y Recipient FS.

## Declaration of competing interest

The authors declare that they have no known competing financial interests or personal relationships that could have appeared to influence the work reported in this paper.
